# Exercise Alters Gait Pattern but Not Knee Load in Patients with Knee Osteoarthritis

**DOI:** 10.1155/2016/7468937

**Published:** 2016-09-20

**Authors:** Ssu-Yu Chang, Yi-Jia Lin, Wei-Chun Hsu, Lin-Fen Hsieh, Yuan-Hsiang Lin, Chao-Chin Chang, You-Cai Chou, Li-Fei Chen

**Affiliations:** ^1^Department of Physical Medicine and Rehabilitation, Shin Kong Wu Ho-Su Memorial Hospital, Taipei 111, Taiwan; ^2^Graduate Institute of Biomedical Engineering, National Taiwan University of Science and Technology, Taipei 106, Taiwan; ^3^National Defense Medical Center, Taipei 114, Taiwan; ^4^Department of Electronic and Computer Engineering, National Taiwan University of Science and Technology, Taipei 106, Taiwan; ^5^Graduate Institute of Applied Science and Technology, National Taiwan University of Science and Technology, Taipei 106, Taiwan

## Abstract

Six female patients with bilateral medial knee OA and 6 healthy controls were recruited. Patients with knee OA received a 6-week physiotherapist-supervised and home-based exercise program. Outcome measures, including the Western Ontario and McMaster Universities Arthritis Index and Short Form-36 Health Survey as well as objective biomechanical indices were obtained at baseline and follow-up. After treatment, no significant difference was observed in the knee abductor moment (KAM), lever arm, and ground reaction force. We, however, observed significantly improved pain and physical function as well as altered gait patterns, including a higher hip flexor moment and hip extension angle with a faster walking speed. Although KAM was unchanged, patients with bilateral knee OA showed an improved walking speed and altered the gait pattern after 6 weeks of supervised exercise. This finding suggests that the exercise intervention improves proximal joint mechanics during walking and can be considered for patients with bilateral knee OA. Non-weight-bearing strengthening without external resistance combined with stretching exercise may be an option to improve pain and function in individuals with OA who cannot perform high resistance exercises owing to pain or other reasons.

## 1. Introduction

Because of the aging of the population, the prevalence of symptomatic knee osteoarthritis (OA), a common degenerative joint disease in the elderly population, has increased [1]. Approximately 10% of people aged more than 55 years have painful disabling knee OA, one-quarter of whom are severely disabled [2]. Patients with knee OA may experience pain, muscle weakness, limited range of motion, deformity, and stiffness [3, 4], causing abnormal gait patterns [5, 6] and reduced health-related quality of life [4]. Treatment of knee OA includes analgesia and nonsteroidal anti-inflammatory drugs, intra-articular hyaluronic acid injection, transcutaneous electric nerve stimulation, acupuncture, and insoles [4, 7, 8]. However, these treatments do not address musculoskeletal strength and flexibility. Studies have reported the therapeutic benefits of exercise on OA symptoms; however, some of these studies have only been based on data from subjective evaluations [9, 10], and quantitative analyses of joint mechanics are limited.

Therapeutic exercise not only reduces OA symptoms but also delays disease progression and is thus beneficial to patients with knee OA [10, 11]. The duration of exercise in studies on the effect of exercise ranges from 4 [11] to 12 weeks [9, 12–14]. Because quadriceps muscle weakness is a frequent finding among patients with knee OA [15] and is associated with pain intensity, physical dysfunction, and decreased function [15, 16], quadriceps strengthening is commonly prescribed for OA knees because it reduces pain and improves function [12, 14]. Although evidence exists that quadriceps strengthening benefits patients with knee OA, increased quadriceps strengthening alone may increase compression forces across the knee joint [17]. An exercise regimen that includes both strengthening and stretching may prevent this possible side effect. However, few studies have evaluated the effect of strengthening exercises on muscle groups other than the quadriceps [11, 13, 18, 19]. Apart from strengthening the lower extremity muscle groups, stretching is an essential component in the clinical intervention for knee OA; however, evidence of the beneficial effect of stretching is limited [11, 19]. Therefore, the effect of exercise that includes both strengthening and stretching in the sagittal and frontal planes on improving the gait pattern remains unclear for patients with knee OA.

An increased knee abductor moment (KAM) is strongly correlated with increased compressive medial compartment load [20], knee pain, OA severity, and rate of progression [21]. KAM is therefore used as a biomechanical index for evaluating the efficacy of interventions, such as orthoses and exercise, in people with medial knee OA [11–14, 18, 19]. Some studies have reported that exercise does not reduce KAM [12–14, 18, 19]; however, Thorp et al. [11] showed that KAM decreased by 9% after 4 weeks of exercise. KAM is primarily composed of the ground reaction force (GRF) and lever arm in the frontal plane; the mechanism by which KAM changes after exercise, particularly for exercises affecting factors influencing the GRF and its lever arm, has also not been reported in the aforementioned studies [11–14, 18, 19]. Thus, including the GRF and its lever arm available at the knee joint as biomechanical variables may aid in understanding the contribution of KAM to the effect of exercise on knee OA.

The quadriceps and hamstrings, which act primarily in the sagittal plane, probably generate minor effects in the frontal plane; thus, targeting hip abductor musculature may be another biomechanically based exercise option for medial knee OA [11]. This concept was proven in a pilot study, in which a training regimen that emphasized hip abductor musculature and conventional quadriceps and hamstring training reduced KAM during walking, with a 78% reduction in pain in patients with knee OA. However, Bennell et al. [13] found that hip strengthening reduced symptoms and contralateral pelvic drop but not knee load. Factors reducing KAM include lower gait speed [22], stride length [23], and pain [24] and a higher toe-out angle [25]. KAM is also reduced by hip-knee-ankle alignment [25], which is influenced by the flexibility of 2-joint muscles related to the knee joint (rectus femoris, hamstring, tensor fasciae latae, and calf). Moreover, muscle groups, including hip abductors, tensor fasciae latae, quadriceps, lateral hamstrings, and lateral gastrocnemius, counteract KAM in healthy adults [26] and patients with knee OA [24]. Although the causal relationship between these muscle groups and KAM has been established, few studies have investigated the effects of exercise for the aforementioned muscle groups on KAM in patients with knee OA [11, 19].

Because alteration in knee joint mechanics because of a disease and/or intervention may cause biomechanical changes in the adjacent joints, changes in joint mechanics at the hip and ankle are also crucial to gait improvement. Similar to Bennell et al. [12], Lim et al. [14] and Thorstensson et al. [19] only observed KAM; however, few studies have focused on this parameter. Therefore, the purpose of the current study was to investigate the effect of exercise on objective biomechanical indices, including 3-dimensional joint mechanics of lower extremities, during gait in patients with bilateral medial knee OA. We hypothesized that a 6-week supervised lower extremity exercise program that emphasizes strengthening at both hip and knee muscles and 2-joint muscle flexibility around the knee alters gait patterns during walking with improved physical function and decreased pain in patients with knee OA.

## 2. Methods

### 2.1. Participants

In the current study, 6 patients with medial knee OA were recruited by a physician (LFH) and were included in the OA group. These patients met the following inclusion criteria: (a) bilateral medial knee OA, (b) Kellgren/Lawrence grade [27] 2 or 3 at the bilateral knee, and (c) ability to walk independently. Patients were excluded if they had (a) received treatment, such as surgery, in the past 6 months, injections, or foot orthoses and (b) patients with DM, other neuromusculoskeletal disorders, visual impairment, or cognitive dysfunction that might disturb gait. Six age- and sex-matched healthy controls were recruited, all of whom had normal or corrected-to-normal vision and no neuromusculoskeletal pathology. The present study was approved by the human research ethics committee of our institute (20110802R). Written informed consent was obtained from all participants.

### 2.2. Descriptive Measures

The Western Ontario and McMaster Universities Arthritis Index (WOMAC) was used to assess symptomatic severity in patients in the OA group. The WOMAC is a disease-specific, self-administered questionnaire for patients with knee OA that is composed of 3 dimensions (with 24 items): pain (5 items), stiffness (2 items), and physical function (17 items). The results are scored on a 0–20 scale for pain, 0–8 scale for stiffness, and 0–68 scale for physical function. Higher scores indicate a higher degree of pain, stiffness, or physical dysfunction. The quality of life of patients in the OA group was measured using the 36-item Short Form-36 Health Survey (SF-36) [28], which is composed of 8 subscales: physical functioning (PF), role-physical [11], bodily pain (BP), general health (GH), vitality (VT), social functioning (SF), role-emotional (RE), and mental health (MH). Each subscale is scored from 0 to 100, and higher scores indicate superior health [28]. We used the Chinese version of the SF-36, for which psychometric properties have been established.

### 2.3. Exercise Program

The objective of the exercise program was to improve lower extremity strength and flexibility (Table 1). During the 6-week exercise program, each participant underwent a total of 6–12 training sessions under the supervision of a senior physical therapist. In each 1-hour supervised session, the therapist ensured the performance and exercise intensity of the muscle stretching and strengthening regimen. Patients also undertook home-based training sessions during this 6-week period. All exercises were repeated in 3 sets: 10 repetitions with 10 seconds bilaterally as tolerated by patients. Pain coping strategies were adopted to ensure that increased symptoms did not persist until the next exercise set of the day. The logbook supplied to participants performing exercises contained detailed instructions on the exercises. To monitor compliance, participants recorded the number of exercises performed each day in a diary. The compliance rate was deemed 100% if the participant had completed 5 days of exercises per week over 6 weeks (a total of 90 exercise sessions). Adherence of at least 75% is necessary to be included in the data analysis.

### 2.4. Gait Analysis

The gait of patients was analyzed at baseline and at 6 weeks after the completion of the exercise program (follow-up). Patients walked at a self-selected pace on an 8 m walkway while infrared retroreflective markers were attached to the posterior superior iliac spine, anterior superior iliac spine, greater trochanters, midthighs, medial and lateral femoral epicondyles, tibial tuberosities, fibular heads, medial and lateral malleoli, heels, navicular tuberosities, and 2nd metatarsal bases [8]. Three-dimensional marker trajectories and the GRF were captured using a 6-camera motion analysis system (Vicon MX 13+ system) with a sampling rate of 120 Hz and 2 forceplates with a sampling rate of 1080 Hz.

For dynamic analysis, the pelvis-leg apparatus was modeled as a 7-link system, with each link embedded with an orthogonal coordinate system. Following the recommendation of the International Society of Biomechanics, the positive *x*-axis was directed anteriorly, the positive *y*-axis superiorly, and the positive *z*-axis to the right [29]. The rotational movements of the joints of the lower extremities were described using the Cardanic rotation sequence (*z-x-y*) [30]. By performing inverse dynamics, intersegmental internal moments at the joints of the lower extremities were calculated using the measured GRF and kinematic data, in which the internal moments were normalized to body weight and leg length. The mass, center of mass, and moment of inertia of each body segment were obtained using Dempster's coefficients [31]. The frontal GRF was also calculated as the resultant force vector of the vertical and M/L component of the GRF, and the corresponding lever arm available at the knee was calculated as the perpendicular distance between the frontal GRF and the knee joint center. Gait variables, including the walking speed, stride time, and step length, were also obtained.

### 2.5. Objective Biomechanical Indices

The maximal value of KAM during early stance phase was defined as the first peak KAM. The maximal value of KAM during late stance phase was defined as the second peak KAM. The peaks of KAM during early (first peak at P1) and late stance phases (second peak at P2) and the corresponding magnitude of the frontal GRF and its lever arm available at the knee were extracted. The joint angles and moments at the hip, knee, and ankle joints when the first and second peak KAM occurred were also extracted for subsequent statistical analysis.

### 2.6. Statistical Analyses

A Wilcoxon test was used to investigate the effect of 6-week exercise on SF-36 and WOMAC scores and on all calculated biomechanical variables at baseline and follow-up. A Mann-Whitney* U* test was used to investigate the differences between patients with OA and controls at baseline and follow-up. All statistical analyses were performed using SPSS (version 17, SPSS Inc., Chicago, USA). All significance levels were set at *α* of 0.05.

## 3. Results

The mean percentage of home-based exercise sessions completed was 84%. None of the participants received medication during the evaluation and training periods. Only one participant sought a cointervention (Modality Therapist), and the data of this patient were excluded from statistical analysis. After the 6-week exercise program, the results of SF-36 showed significantly lower physical functioning (*Pt* < 0.05) and pain (*Pt* < 0.05) and unchanged scores for RP, BP, VT, SF, RE, and MH (physical role limitations: *Pt* > 0.05; general health perceptions: *Pt* > 0.05; energy/vitality: *Pt* > 0.05; social functioning: *Pt* > 0.05; and mental health: *Pt* > 0.05). After treatment, the results of WOMAC showed unchanged scores for stiffness, but significantly lower scores for pain and physical function were found compared with those obtained before treatment (pain: *Pt* = 0.03; physical function: *Pt* = 0.002; and stiffness: *Pt* > 0.05).

### 3.1. Objective Biomechanical Indices

#### 3.1.1. Temporal-Spatial Parameters

Baseline evaluation of temporal-spatial parameters of walking revealed no significant between-group differences in step length and step width, and the OA group displayed a longer walking time and a significantly slower walking speed (*Pg* = 0.03, Table 2). After 18 exercise sessions, no significant difference was observed in any of the calculated temporal-spatial parameters between the groups (*Pg* > 0.05, Table 2). For the treatment effect in the OA group, the comparison between pre- and postevaluation time revealed a faster walking speed.

#### 3.1.2. Joint Mechanics Variables

**Figure 1:**
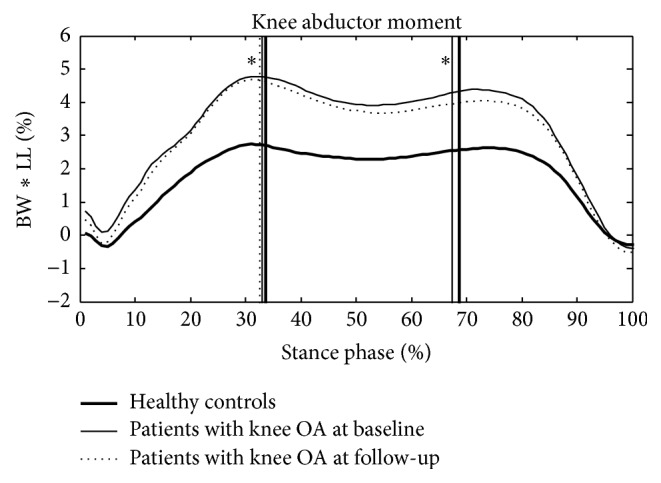
Ensemble-averaged patterns of KAM in healthy controls (thick solid lines) and patients with knee OA at baseline (thin solid lines) and follow-up (thin dashed lines). Vertical lines indicate the time points when the first and second peak KAM occurred; %BW *∗* LL: percentage of leg length multiplied by body weight; ^⁎^significant group effects *P* < 0.05.

For the group effect at baseline and follow-up, a significant difference was observed in the first KAM (Figure 1 and Table 3) and its lever arm (Table 3), whereas no difference was identified in the frontal GRF (Table 3), and no group effect was observed in the second KAM, its lever arm, and the frontal GRF (Table 4). For the treatment effect in the OA group, no significant difference was observed in the first and second KAM and their lever arms and the GRF between baseline and follow-up evaluation (Tables 3 and 4).

Baseline evaluation revealed no between-group difference in the hip, knee, and ankle joint angles in the frontal plane or in the hip and ankle joint moments in the sagittal plane at P1 (Table 3) and P2 (Table 4). Follow-up evaluation demonstrated that the OA group showed a significantly increased hip external rotation moment when the first peak KAM occurred (Table 3) and significantly increased the hip extension angle and hip flexor moment at P2 (Table 4). For the treatment effect in the OA group, the hip external rotator moment significantly increased when the first peak KAM occurred (Table 3), and the hip external rotation angle, hip extension angle, and flexor moment significantly increased when the second peak KAM occurred after 18 exercise sessions (Table 4).

## 4. Discussion

This study evaluated the effect of a 6-week supervised exercise program that included strengthening and stretching in patients with bilateral medial knee OA. The results showed that specific gait adaptations, including a significantly increased hip flexor moment and faster walking speed, were observed after the intervention, even though knee loading was not reduced compared with that at baseline. Significantly lower scores of physical function in both SF-36 and WOMAC as well as decreased pain suggested that patients with knee OA responded favorably to the exercise program used in the current study.

Performing resistance lower extremity exercises can aggravate OA knee symptoms [32, 33]. Although several studies have included stretching in conventional therapeutic exercise programs for patients with knee OA, these studies have not investigated the effects of stretching exercise [12–14, 18]. Using an exercise program that includes strengthening and stretching for clinically managing patients with knee OA is encouraged according to the findings of reciprocal positive effects between 2 common complications of knee OA, namely, pain and dysfunction. The positive effects of exercise on pain are multifactorial and thus difficult to delineate clearly. A previous study showed that pain improvement was not related specifically to one type of strengthening exercise but was mainly attributed to a general increase in physical activity following a home-based exercise program [34]. Although our patients did not receive medication for pain control, their pain scores improved. The significantly improved physical function displayed in the current study is consistent with the finding in a previous study, which demonstrated improved WOMAC functional scores after a home-based strengthening program [13]. The underlying mechanism may have illustrated the possible effect of exercise on the increased of fat and loss of protein linked to chronic inflammation in patients with arthritis [34].

Exercise is routinely prescribed for patients with knee OA. A systematic review reported that a general exercise program for patients with knee OA should include strengthening and stretching [34]. Hence, the effect of an exercise program that includes strengthening and stretching on pain and physical function was determined. The present study found that the peak KAM during walking was unchanged after the exercise intervention, consistent with the findings of previous studies [12–14, 18, 19, 32]. By contrast, Thorp et al. [11] demonstrated that peak KAM decreased after a 4-week exercise intervention specifically targeting hip abductor strength as well as conventional quadriceps and hamstring training. Thorstensson et al. [19] also showed that KAM was not significantly reduced during gait but was significantly reduced during one-leg rising after the completion of an 8-week strengthening and functional exercise program, suggesting that the peak KAM is more likely to be altered during a more demanding task than during gait. Lim et al. [14] found no significant change in KAM in patients with medial knee OA who had varus malalignment (>5 degrees) or neutral alignment (<5 degrees) after quadriceps muscle strengthening. Although strengthening in the frontal plane is necessary to counteract the varus torque and to shift the load from the medial to the lateral compartment [11], this mechanism does not mainly contribute to the reduction in KAM. Because KAM is affected by the GRF and lever arm in the frontal plane, we directly calculated the lever arm of the GRF in the frontal plane in the current study. We found that the higher KAM in the OA group compared with controls was mainly attributed to the increased frontal plane lever arm (Table 3) without increased GRF (Table 3). Evaluation of the treatment effect revealed that the lever arm remained unchanged after the intervention (Table 3) and KAM was unchanged (Table 3). Apart from having reported the commonly reported first peak KAM during the first half of the stance phase, the current study was the first to extract the second peak KAM during the propulsive phase of walking to investigate the effect of exercise on gait patterns in patients with knee OA. According to the unchanged first and second peak KAM, we demonstrated that the biomechanical index of KAM tends not to be influenced by the exercise intervention alone during gait.

For the temporal-spatial variables, the comparison between pre- and postevaluation time in the current study revealed an increased step length and walking speed (Table 2). At baseline, longer walking time (Table 2) and slower walking speed (Table 2) were observed in the OA group compared with controls. However, these findings were not significant at follow-up (Table 2), indicating that the walking time and walking speed were similar to those in controls after the exercise intervention. The significantly greater speed at follow-up may be associated with the relief of pain after treatment through increased hip extension angle and increased flexor moment (Table 4). Previous studies have found significantly increased walking speed after a 3-month exercise intervention in patients with knee OA [12, 33], whereas the walking speed has been unchanged in other studies [11, 19]. To the best of our knowledge, among studies on the effect of exercise in patients with knee OA, only one study included healthy individuals as a standard reference of gait performance for comparison [18]. As walking speed can be taken as a functionally related parameter during walking [33], these aforementioned results and our finding of the improved walking speed confirm the positive effect of exercise on walking efficiency.

Although harmful KAM was not reduced after 6 weeks of exercise, specific gait adaptations, including an increased hip flexor moment and hip extension angle, were found at P2 (Table 4), which may aid in the propulsive performance required during the following push-off phase. During the terminal stance phase when the body weight is shifted forward to place the limb into the trailing posture, the body vector moves posteriorly to the hip joint, and the thigh is pulled into extension. Consistent with studies investigating joint mechanics at the hip to evaluate the effect of exercise in patients with knee OA [11, 13], exercise did not alter hip abductor moments during walking in this study [11, 13]. We also observed a higher hip extension angle at P2, consistent with the finding of Thorp et al. [11]; they reported a higher excursion of the hip flexion/extension angle with an unchanged hip abductor moment after the exercise intervention. Few recent studies have included 2-joint muscle stretching exercise [11, 19] and strengthening exercise at hip and knee joints [11, 12, 19], which were included in our study. Thus, our exercise program was expected to affect proximal part joint mechanics regarding hip angles and moments. Stretching exercise may also increase joint excursion and affect muscular activities, which may be associated with joint moments. Notably, when the second peak KAM occurred, the hip extension angle and hip flexor moment increased after our exercise program, which included strengthening and stretching the proximal part of lower extremities. This increased hip extension and flexor moment may be helpful during this preswing phase according to the tension-length relationships. In other words, when muscles contract at longer lengths, tension partly depends on the passive stretching of the connective tissue within the muscle, acting in parallel with the active force generated by the muscle fibers themselves. Thus, when muscles, including iliopsoas and rectus femoris, are stretched more because of increased hip extension, muscular contraction may be maximized, and gait performance, such as gait speed, may be improved as a consequence, as indicated in the current study (Table 2).

Evidence has indicated that the level of patient self-reliance required to sustain the exercise program through a home-based program determined the exercise effect [34]. Hence, in this study, monitoring sheets were used for the exercise program to ensure a sufficient level of patient self-reliance to sustain exercise, which may have been another contributor to the identified clinical improvement. A systematic review of the effect of the exercise type on pain and disability in patients with knee OA suggested that optimal exercise should be supervised and performed 3 times per week, and this minimum exercise frequency is crucial for pain relief and disability reduction [3]. Our daily exercise frequency was based on another previous study that evaluated the biomechanical effects of muscle training in patients with medial knee OA [11]. High patient compliance (88%) was obtained in this study. We found that a 6-week supervised home-based exercise intervention 5 days per week can improve pain, physical function, and walking speed and alter the gait pattern in patients with knee OA.

This study had several limitations. First, the lack of a control OA group may have limited the validity of the conclusions of the current study. Nevertheless, the biomechanical endpoints were nonsubjective, and these are known to be stable over time in untreated individuals; hence, the objective gait adaptation was likely attributed to the biomechanical exercise intervention rather than to a placebo effect. The current findings showed some significant changes in gait after 6 weeks of exercise. For the first time in the literature, the observed gait adaptation indicated the effect of exercise on 3-dimensional joint mechanics at P1 and P2 and at the lever arm of the GRF at the knee in patients with knee OA, comprehensively illustrating the gait pattern before and after exercise. Additional studies are required to determine how long the acquired gait patterns can be maintained if exercise is discontinued. Future studies should use larger sample sizes. Moreover, because we only included level walking when assessing gait patterns, additional studies that include more challenging tasks such as staring and obstacle crossing should be conducted. Nevertheless, based on the various exercise types proposed in the literature, the current study was the first to provide beneficial evidence of non-weight-bearing strengthening without external resistance combined with stretching exercise for patients with knee OA.

## 5. Conclusions

The effect of exercise on walking was investigated in patients with bilateral medial knee OA. Although peak KAM was unchanged, our 6-week exercise program improved pain and physical function and altered gait patterns, producing results such as a higher hip flexor moment and hip extension angle with faster walking speed. This finding indicated that the exercise program had positive effects on patients with bilateral medial knee OA. Therefore, a supervised exercise program that monitors the overall lower extremity muscular conditions and includes strengthening and stretching 5 days per week over 6 weeks may generate biomechanical benefits with clinical improvement. Non-weight-bearing strengthening without external resistance combined with stretching exercise improves pain and function with a higher walking speed and altered gait patterns at the hip in individuals with OA. Such an exercise program should thus be encouraged in patients with bilateral medial knee OA.

## Figures and Tables

**Table 1:** Exercise program.

Strengthening exercises	Perform daily	
	Repetition: hold for 10 s × 10 repetitions and repeat 3 times	
Exercise	Performance	Clinical observation
Quadriceps strengthening (hip flexor)	In the supine position, bend the contralateral knee in flexion; raise the exercise limb with the knee in full extension to the height of the contralateral flexed knee.	Avoid lumbar hyperlordosis with back symptoms.
Gluteus maximus strengthening (hip extensor)	In the prone position, raise the exercise limb with the knee in full extension to the ceiling.	Avoid lumbar hyperlordosis with back symptoms.
Gluteus medius strengthening (hip abductor)	In the lateral lying position, bend the knee and hip of the lower leg and raise the upper leg, keeping the knee straight.	Assure that hip joint remains in slight extension and without rotation.
Adductor magnus strengthening (hip adductor)	In the lateral lying position, bend the knee and hip of the upper leg in front and raise the low leg, keeping the knee straight.	
Quadriceps strengthening (knee extensor)	Sit with legs over the edge of the table, allowing the knees to flex to 90°, and slowly extend the knee to full extension and then return the knee to the flexed position.	

Muscle flexibility exercises	Perform daily	
	Repetition: hold for 10 s × 10 repetitions and repeat 3 times	
Exercise	Performance	Clinical observation

Standing calf stretch	Stand with the heel of the foot on the ground, with toes pointing straight ahead. Lean forward until the calf is stretched. Use both arms for support against a wall.	
Supine hamstring muscle stretch	In the supine position, flex the ipsilateral hip to 90°. Straighten the knee with a strap under the foot. Grasp the strap by using both hands and pull until the posterior thigh and calf are stretched.	If radicular symptoms are produced, then reduce the intensity of the stretch.
Prone quadriceps femoris muscle stretch	In the prone position, extend both hips and knees. Place a strap around the ipsilateral ankle. Grasp the strap by using both hands and bend the knee until the anterior thigh is stretched.	Avoid lumbar hyperlordosis with back symptoms and hamstring cramping.

Muscle flexibility exercises	Perform daily	
	Repetition: hold for 90 s at each point and repeat 3 times daily	
Exercise	Performance	Clinical observation

Lateral lying tensor fasciae latae massage	In the lateral lying position, place a tennis ball on the lateral surface of the thigh to produce ischemia compression.	Pressure as pain tolerance

**Table 2:** Temporal-spatial parameters.

	Group	Evaluation time				Group effect (baseline)		Group effect (follow-up)		Treatment effect	
		Baseline		Follow-up		Mean difference of 95% CI	*Pg* ^*∗*^	Mean difference of 95% CI	*Pg* ^*∗*^	Mean difference of 95% CI	*Pt* ^†^
		Mean	SD	Mean	SD						
Walking time (sec)	Control	1.60	0.10			0~0.18	0.05^*∗*^	0.04~0.5	0.19	N/A	N/A
	OA	1.79	0.22	1.75	0.32					−0.1~0.2	0.49

Walking speed (m/sec)	Control	0.93	0.06			0~0.18	0.03^*∗*^	0.04~0.5	0.19	N/A	N/A
	OA	0.81	0.11	0.87	0.17					−0.1~0	0.21

Step length (% LL)	Control	60.52	3.85			0.15~0.65	0.29	0.15~0.6	0.35	N/A	N/A
	OA	62.65	4.71	62.90	4.53					−3.4~2.9	0.85

Step width (% LL)	Control	10.50	3.06			0.15~0.65	0.29	0.04~0.5	0.24	N/A	N/A
	OA	8.58	3.13	8.58	2.07					−2.4~2.4	1.00

Foot progression angle	Control	6.95	3.83			0.43~0.91	0.64	0.6~1	0.72	N/A	N/A
	OA	7.82	7.24	8.32	6.62					−3.7~2.8	0.71

^*∗*^ *P* values for between-group comparisons at different evaluation times (baseline and follow-up); ^*∗*^ *Pg* < 0.0*5.*

^†^ *P* values for within-group comparisons between baseline and follow-up to evaluate the treatment effect; ^†^ *Pt* < 0.05.

**Table 3:** Joint mechanics at 1st peak knee abductor moment.

	Group	Evaluation time				Group effect (baseline)		Group effect (follow-up)		Treatment effect	
		Baseline		Follow-up		Mean difference of 95% CI	*Pg* ^*∗*^	Mean difference of 95% CI	*Pg* ^*∗*^	Mean difference of 95% CI	*Pt* ^†^
		Mean	SD	Mean	SD						
Frontal GRF (% BW)	Control	95.81	5.64			0.81~1	0.81	0.6~1	0.72	N/A	N/A
	OA	97.54	2.51	97.17	3.76					−3.5~4.2	0.82

Frontal lever arm (% LL)	Control	2.95	0.91			0~0.18	0.02^*∗*^	0~0.2	0.06	N/A	N/A
	OA	4.56	1.29	4.37	1.02					−0.5~0.9	0.53

Ankle In(+)/Ev(−) angle	Control	−1.43	4.38			0.35~0.85	0.56	0.35~0.8	0.56	N/A	N/A
	OA	−3.08	2.36	−2.68	2.37					−2.4~1.6	0.62

Ankle IR(+)/ER(−) angle	Control	0.67	3.36			0.6~1	0.72	0.04~0.5	0.24	N/A	N/A
	OA	−0.46	1.97	−1.44	2.24					−0.4~2.3	0.12

Ankle Do(+)/Pl(−) angle	Control	3.46	4.52			0.15~0.65	0.41	0.15~0.6	0.29	N/A	N/A
	OA	1.37	3.13	0.73	2.75					−1.2~2.5	0.42

Ankle In(−)/Ev(+) moment	Control	−0.19	0.62			0.15~0.65	0.29	0.43~0.9	0.64	N/A	N/A
	OA	−0.32	0.11	−0.20	0.27					−0.5~0.2	0.43

Ankle IR(−)/ER(+) moment	Control	−0.99	0.68			0.21~0.72	0.48	0.15~0.6	0.35	N/A	N/A
	OA	−1.30	0.45	−1.31	0.39					−0.2~0.3	0.95

Ankle Do(−)/Pl(+) moment	Control	2.97	2.39			0~0.31	0.08	0.04~0.5	0.16	N/A	N/A
	OA	4.21	1.04	4.12	0.89					−0.7~0.9	0.77

Hip Add(+)/Abd(−) angle	Control	8.29	2.01			0.35~0.85	0.56	0.04~0.5	0.24	N/A	N/A
	OA	8.93	2.04	7.29	1.30					−0.5~3.8	0.10

Hip IR(+)/ER(−) angle	Control	−3.24	3.56			0~0.4	0.1	0.43~0.9	0.64	N/A	N/A
	OA	−0.23	2.27	−4.19	2.01					−0.1~8	0.05^†^

Hip Flex (+)/Ex(−) angle	Control	4.05	23.17			0.82~1	1	0.82~1	1.00	N/A	N/A
	OA	8.75	6.27	9.87	5.55					−5.7~3.4	0.55

Hip Add(−)/Abd(+) moment	Control	9.03	5.25			0.04~0.49	0.19	0.15~0.6	0.41	N/A	N/A
	OA	12.28	0.94	11.75	1.25					−0.8~1.9	0.36

Hip IR(−)/ER(+) moment	Control	0.41	0.64			0.04~0.49	0.19	0~0.2	0.00^*∗*^	N/A	N/A
	OA	0.96	0.82	2.34	0.56					−2.6~−0.1	0.04^†^

Hip Flex (−)/Ex(+) moment	Control	1.24	1.13			0.04~0.49	0.16	0.15~0.6	0.35	N/A	N/A
	OA	2.41	1.85	2.19	1.78					−2.3~2.7	0.83

Knee Add(+)/Abd(−) angle	Control	−0.50	3.33			0.35~0.85	0.56	0.43~0.9	0.64	N/A	N/A
	OA	0.15	2.76	−0.17	2.08					−1.2~1.9	0.62

Knee IR(+)/ER(−) angle	Control	−0.19	1.83			0.35~0.85	0.56	0~0.2	0.01^*∗*^	N/A	N/A
	OA	−0.92	2.36	−2.85	1.86					−1.1~4.9	0.16

Knee Flex (+)/Ex(−) angle	Control	6.86	23.12			0.15~0.65	0.35	0.15~0.6	0.35	N/A	N/A
	OA	9.68	6.43	9.99	5.66					−3.7~3.1	0.83

Knee Add(−)/Abd(+) moment	Control	2.85	1.77			0~0.18	0.01^*∗*^	0~0.2	0.01^*∗*^	N/A	N/A
	OA	4.96	1.14	4.88	0.81					−0.5~0.7	0.74

Knee IR(−)/ER(+) moment	Control	−0.35	0.24			0~0.18	0.01^*∗*^	0~0.2	0.03^*∗*^	N/A	N/A
	OA	−0.71	0.26	−0.70	0.29					−0.2~0.2	0.87

Knee Flex(−)/Ex(+) moment	Control	3.02	2.26			0.81~1	0.81	0.21~0.7	0.48	N/A	N/A
	OA	3.18	0.97	3.63	0.76					−1.8~0.9	0.44

^*∗*^ *P* values for between-group comparisons at different evaluation times (baseline and follow-up); ^*∗*^ *Pg* < 0.05.

^†^ *P* values for within-group comparisons between baseline and follow-up to evaluate the treatment effect; ^†^ *Pt* < 0.05.

**Table 4:** Joint mechanics at 2nd peak knee abductor moment.

	Group	Evaluation time				Group effect (baseline)		Group effect (follow-up)		Treatment effect	
		Baseline		Follow-up		Mean difference of 95% CI	*Pg* ^*∗*^	Mean difference of 95% CI	*Pg* ^*∗*^	Mean difference of 95% CI	*Pt* ^†^
		Mean	SD	Mean	SD						
Frontal GRF (% BW)	Control	97.30	6.51			0.81~1	0.81	0.43~0.9	0.64	N/A	N/A
	OA	97.23	3.98	97.12	2.64					−4~4.2	0.95

Frontal lever arm (% LL)	Control	2.22	0.65			0.04~0.49	0.16	0~0.4	0.10	N/A	N/A
	OA	3.43	2.07	3.21	1.58					−0.8~1.2	0.60

Ankle In(+)/Ev(−) angle	Control	−2.00	3.41			0.43~0.91	0.64	0.35~0.8	0.56	N/A	N/A
	OA	−2.61	3.29	−1.23	2.37					−3.6~0.8	0.17

Ankle IR(+)/ER(−) angle	Control	2.63	3.25			0.21~0.72	0.48	0.81~1	0.81	N/A	N/A
	OA	1.25	2.66	1.24	2.29					−1.7~1.7	1.00

Ankle Do(+)/Pl(−) angle	Control	8.13	2.89			0.15~0.65	0.29	0.04~0.5	0.19	N/A	N/A
	OA	6.94	3.50	5.66	3.66					0.35~2.2	0.02^†^

Ankle In(−)/Ev(+) moment	Control	−0.31	0.97			0.21~0.72	0.48	0.43~0.9	0.64	N/A	N/A
	OA	−0.57	0.52	−0.59	0.81					−2.2~3.5	0.58

Ankle IR(−)/ER(+) moment	Control	−3.20	2.22			0.15~0.65	0.35	0.04~0.5	0.16	N/A	N/A
	OA	−4.17	1.48	−4.28	0.78					−1.3~1.5	0.84

Ankle Do(−)/Pl(+) moment	Control	9.77	6.03			0.6~1	0.72	0.6~1	0.72	N/A	N/A
	OA	12.73	1.66	12.75	1.55					−2.3~2.2	0.98

Hip Add(+)/Abd(−) angle	Control	6.55	2.49			0.35~0.85	0.56	0.81~1	0.81	N/A	N/A
	OA	6.83	3.30	6.55	1.96					−3.5~4.1	0.86

Hip IR(+)/ER(−) angle	Control	0.60	2.53			0.04~0.49	0.16	0.21~0.7	0.48	N/A	N/A
	OA	2.90	3.43	−0.58	1.49					0.2~6.8	0.04^†^

Hip Flex(+)/Ex(−) angle	Control	−5.90	21.37			0.15~0.65	0.84	0.81~1	0.03^*∗*^	N/A	N/A
	OA	−5.75	3.24	−7.10	3.66					−0.6~3.3	0.04^†^

Hip Add(−)/Abd(+) moment	Control	8.25	4.79			0.15~0.65	0.29	0.15~0.6	0.41	N/A	N/A
	OA	11.33	1.97	10.60	1.13					−0.6~2	0.21

Hip IR(−)/ER(+) moment	Control	−1.19	0.86			0.35~0.85	0.56	0~0.2	0.05^*∗*^	N/A	N/A
	OA	−0.92	0.72	0.00	0.48					−2~0.1	0.07

Hip Flex(−)/Ex(+) moment	Control	−1.70	1.61			0.15~0.65	0.35	0~0.2	0.03^*∗*^	N/A	N/A
	OA	−2.58	1.34	−3.62	1.23					0.77~1.3	0.00^†^

Knee Add(+)/Abd(−) angle	Control	−0.20	3.33			0.43~0.91	0.64	0.81~1	0.91	N/A	N/A
	OA	−0.59	3.10	−0.88	1.97					−1.4~2	0.67

Knee IR(+)/ER(−) angle	Control	0.60	3.00			0.21~0.72	0.48	0.81~1	0.91	N/A	N/A
	OA	2.00	3.46	0.31	2.71					−2.1~5.5	0.31

Knee Flex (+)/Ex(−) angle	Control	3.76	22.84			0.21~0.72	0.48	0.35~0.8	0.56	N/A	N/A
	OA	8.47	8.33	7.58	6.89					−2.6~4.4	0.54

Knee Add(−)/Abd(+) moment	Control	2.79	1.68			0~0.19	0.06	0~0.3	0.08	N/A	N/A
	OA	4.61	1.51	4.27	1.07					−0.6~1.2	0.37

Knee IR(−)/ER(+) moment	Control	−1.01	0.66			0~0.4	0.1	0~0.3	0.08	N/A	N/A
	OA	−1.69	0.70	−1.61	0.42					−0.6~0.5	0.72

Knee Flex (−)/Ex(+) moment	Control	−0.77	1.83			0.81~1	0.81	0.15~0.6	0.29	N/A	N/A
	OA	−0.41	0.65	−0.12	1.06					−1.7~1.1	0.62

^*∗*^ *P* values for between-group comparisons at different evaluation times (baseline and follow-up); ^*∗*^ *Pg* < 0.05.

^†^ *P* values for within-group comparisons between baseline and follow-up to evaluate the treatment effect; ^†^ *Pt* < 0.05.
